# Microwave-Assisted Synthesis of Nanoporous Aluminum-Based Coordination Polymers as Catalysts for Selective Sulfoxidation Reaction

**DOI:** 10.3390/polym9100498

**Published:** 2017-10-11

**Authors:** Madhan Vinu, Wei-Cheng Lin, Duraisamy Senthil Raja, Jeng-Liang Han, Chia-Her Lin

**Affiliations:** Department of Chemistry, Chung Yuan Christian University, Chung Li, Taoyuan 32023, Taiwan; vinuchem06@gmail.com (M.V.); jack760425@hotmail.com (W.-C.L.); senthilraja1985@gmail.com (D.S.R.)

**Keywords:** aluminum, heterogeneous catalysis, oxidation of sulfides

## Abstract

A series of aluminum-based coordination polymers or metal–organic frameworks (Al–MOFs), i.e., DUT-4, DUT-5, MIL-53, NH_2_-MIL-53, and MIL-100, have been facile prepared by microwave (MW)-assisted reactions and used as catalysts for selective sulfoxidation reactions. The MW-assisted synthesis drastically reduced the reaction time from few days to hours. The prepared MOFs have smaller and uniform particle sizes and better yield compared to conventional hydrothermal method. Furthermore, the Al–MOFs have been successfully demonstrated as catalysts in oxidation reaction of methyl phenyl sulfide with H_2_O_2_ as oxidant, even under mild conditions, with more than 95% conversion.

## 1. Introduction

Porous coordination polymers (PCPs) or metal–organic frameworks (MOFs) containing abundant metals have fascinated the academic as well as industrial based researchers around the world. In this regard, aluminum-based MOFs (Al–MOFs) are considered to be one of the better choices because of their high stability and porosity [1,2,3,4,5]. In addition to the large abundance of Al source, the low cost and less toxic nature of aluminum leads the chemistry of Al based MOFs to be of special interest [6]. In particular, MIL-100 [4], MIL-53 [2] and the isoreticular MOFs of MIL-53, such as DUT-4 [1], DUT-5 [1], and NH_2_-MIL-53 [3], have been investigated for their unique structural properties and potential applications [3,7,8,9]. Hence, our recent research interests are mainly devoted on the synthesis, properties and application studies of nanoporous aluminum MOFs [10,11,12,13,14].

In general, the synthesis of MOFs has been mainly achieved by conventional heating through hydrothermal/solvothermal reactions, which requires several days to weeks [15]. In this context, a new microwave (MW) assisted reaction has been introduced as a more rapid way to reduce the synthesis time with increasing production rate [16]. The homogeneous effects of MW could create a uniform seeding condition, cleaner products, and higher yields [17]. Moreover, it establishes that the MW heating can provide the fast kinetics of crystal nucleation, short reaction times, crystal growth, and high yields in the production of MOFs [18]. Some landmarked MOFs such as MOF-5 [19], HKUST-1 [20], and UiO-66 [21] have been prepared via microwave-assisted synthetic routes. Our group also reported microwave (MW) assisted synthesized calcium and strontium MOFs with selective carbon dioxide adsorption [22].

The use of a green oxidant such as H_2_O_2_ represents an important goal in oxidation reactions since it is non-toxic, cheap and forms water as the only by-product [23]. Furthermore, the selective synthesis of sulfoxides over the fully oxidized sulfones represents another important challenge and, hence, the development of highly efficient catalytic systems for the selective oxidation of sulfides to sulfoxides is of much current interest. Although quite a number of homogeneous and heterogeneous H_2_O_2_-based catalytic systems for the oxidation of sulfides have been reported in the literature [24], most of them have some disadvantages such as the use of excess H_2_O_2_, low chemoselectivity, limited applicability and toxicity of catalysts. To overcome the above issues, MOF/H_2_O_2_ based catalytic systems have now been studied for the selective sulfoxidation reactions [25,26] because MOFs have high surface areas over the other porous material catalysts that can create more catalytic sites, and the tunable porosity of MOFs can provide a special cavity environment for substrates to interact with catalytically active sites which can enhance the activity and selectivity [27].

With this above background, herein, we report the MW-assisted green synthesis of five microporous Al-based MOFs: DUT-4, DUT-5, MIL-53, NH_2_-MIL-53, and MIL-100. The catalytic properties through evaluating the selective catalytic performance of MW-assisted synthesized MOFs on the conversion of methyl phenyl sulfide to methyl phenyl sulfoxide as a model reaction was achieved.

## 2. Experimental Section

### 2.1. Materials and Methods 

Chemicals of reagent grade or better were used as received. MW-assisted reactions were placed in the microwave oven (START D, Milestone, the maximum power of 1200 W, 2.45 GHz, Fatebenefratelli, Sorisole, Italy) with precursor mixtures loaded in a 100 mL Teflon autoclave. The powder X-ray diffraction (PXRD) patterns have been measured using Bruker D8 PHASER powder diffraction instrument (Billerica, MA, USA) with Kα radiation of Cu target as the light source (λ = 0.154056 nm), and silicon plate as the sample stage (holder). For the PXRD measurements, a continuous step-scan mode with step width of 0.030°, equilibrium time of 256 s per step, and the operating voltage of 30 kV and 10 mA have been used. Thermal gravimetric (TG) analyses using a DuPont TA Q50 analyzer (Wilmington, DE, USA), were performed on powder samples under flowing N_2_ with a heating rate of 10 °C/min. Fourier transform infrared (FT-IR) spectra were recorded in the range 400–4000 cm^−1^ on a JASCO FT/IR-460 spectrophotometer using KBr pellets (Oklahoma City, OK, USA). Particle size was analyzed by using 90Plus Particle Size Analyzer from Brookhaven Instruments Corporation (Holtsville, NY, USA). Field-emission scanning electron microscopy (FESEM, Jeol JSM 7600F, Tokyo, Japan) was employed to characterize the morphology; a pinch of finely grained powder was sprinkled gently with a spatula on the copper tape for FESEM. The N_2_ gas sorption isotherms were measured at 77 K using Micromeritics ASAP 2020 adsorption apparatus (Norcross, GA, USA). The ^1^H nuclear magnetic resonance (NMR) signals were measured using BRUKER 300 MHz NMR spectrometer (Billerica, MA, USA).

### 2.2. Synthetic Procedure for Al-MOFs

#### 2.2.1. MW Synthesis of DUT-4

The reaction mixture of Al(NO_3_)_3_·9H_2_O (0.176 g, 0.47 mmol, Merck (98%), Darmstdt, Germany), 2,6-napthalenedicarboxylic acid (H_2_NDC, 0.087 g, 0.40 mmol, Acros (≥97%), Geek, Belgium), and EtOH (10.0 mL, ECHO chemicals (99.5%), Taipei, Taiwan) were placed in the 100 mL Teflon autoclave. Then, the autoclave was placed in MW oven and heated at 120 °C for 46 min. The pale yellow color product was separated from the reaction mixture using centrifugation and washed three times with EtOH solvent followed by dried in an oven at 50 °C. The obtained microcrystalline powdered product, DUT-4(Al), was activated at 190 °C for 12 h under vacuum. Based on the H_2_NDC reagent, the yield of DUT-4(Al) was calculated to be 91%.

#### 2.2.2. MW Synthesis of DUT-5 

DUT-5(Al) was obtained from the reaction mixture of Al(NO_3_)_3_·9H_2_O (0.176 g, 0.47 mmol), 4,4′-biphenyldicarboxylic acid (H_2_BPDC, 0.087 g, 0.36 mmol, Acros (≥97%), Geek, Belgium), and EtOH (10.0 mL). The reaction mixture was heated at 120 °C, for 46 min in MW oven. The product was separated from the reaction mixture using centrifugation and washed three times with EtOH solvent followed by overnight drying in an oven at 50 °C. The obtained creamy white crystalline powder was activated at 190 °C under vacuum. After the activation, the yield of DUT-5(Al) was 76% based on the H_2_BPDC reagent.

#### 2.2.3. MW Synthesis of MIL-53

The reaction mixture of Al(NO_3_)_3_·9H_2_O (0.3752 g, 1.0 mmol), 1,4-benzenedicarboxylic acid (H_2_BDC, 0.0831 g, 0.5 mmol, Merck (≥99%), Darmstdt, Germany) and H_2_O (6.0 mL), was heated at 200 °C, for 83 min. The product was separated from the reaction mixture using centrifugation and washed three times with H_2_O followed by dried overnight in an oven at 50 °C. Activation process: the white crystalline powder was first heated at 330 °C for 3 days under air to remove the unreacted H_2_BDC in the pores of MIL-53(Al) and then heated at 200 °C under vacuum overnight. The yield of the final product was about 69% based on the H_2_BDC reagent.

#### 2.2.4. MW Synthesis of NH_2_-MIL-53 

The reaction mixture of Al(NO_3_)_3_·9H_2_O (0.375 g, 1.0 mmol), 2-aminoterephthalic acid (H_2_BDC-NH_2_, 0.091 g, 0.5 mmol, Alfa (≥97%), Heysham, UK), and H_2_O (6.0 mL), was heated at 200 °C for 52 min. The obtained product was washed three times with H_2_O followed by overnight drying in an oven at 50 °C. The pale yellow powdered product of NH_2_-MIL-53(Al) was activated at 150 °C under vacuum for 12 h. The yield was 73% based on the H_2_BDC-NH_2_ reagent.

#### 2.2.5. MW Synthesis of MIL-100 

The reaction mixture of Al(NO_3_)_3_·9H_2_O (0.664 g, 1.8 mmol), trimesic acid trimethyl ester (Me_3_BTC, 0.378 g, 1.5 mmol, Aldrich (≥97%), St. Louis, MO, USA), H_2_O (5.0 mL), and HNO_3_ (0.3 mL, 4.0 mmol, Merck, Darmstdt, Germany) was heated at 200 °C for 83 min. The product was washed three times with H_2_O followed by overnight drying in an oven at 50 °C. Activation process: the powder was immersed in H_2_O (100 mL) and then stirred at 80 °C for 12 h, the process was repeated for 2 times, and the final creamy white product was heated at 150 °C overnight under vacuum. The yield of activated MIL-100(Al) was 67% based on the Me_3_BTC reagent.

### 2.3. Catalytic Experiment

In a typical experiment, a suspension of our MW-synthesized Al-MOFs catalyst (0.01 mmol) in 5 mL acetonitrile (ACN) was mixed with a solution of methyl phenyl sulfide (1.0 mmol). One equivalent of oxidant, H_2_O_2_ (35% in water, Aldrich (≥97%), St. Louis, MO, USA), was added dropwise and the overall reaction was either stirred at room temperature or heated at 60 °C. The reaction was monitored at different time intervals by ^1^H NMR spectrometer using CDCl_3_ as solvent. In the NMR spectrum, the singlet peak area for methyl proton of the reactant, methyl phenyl sulfide (δ: ~2.4) and the products, methyl phenyl sulfoxide (δ: ~2.7) and methyl phenyl sulfone (δ: ~3.1) were used to calculate the conversion rate and the selectivity. All the NMR spectra are given in Supplementary Materials.

## 3. Results and Discussion

### 3.1. Synthesis

To greenly synthesize the microporous Al-MOFs using MW-assisted hydro-/solvo-thermal reactions, we have first tried the literature procedure with similar reactant ratios and temperature for all the Al-MOFs in which environmentally friendly (green) solvent, either water or ethanol was used instead of toxic dimethylformamide (DMF). The use of water or ethanol as the solvent offers some interesting synthetic advantages such as lower cost, easy availability, nontoxicity, and a green procedure. The MOFs were synthesized by MW-assisted hydrothermal reactions at temperatures between 120 and 210 °C, which led to significant yields (67–91%) within 2 h of reaction time. The final optimized MW-assisted synthetic conditions and the activation process for all the MOFs are given in the experimental section.

### 3.2. Characterization

**Figure 1:**
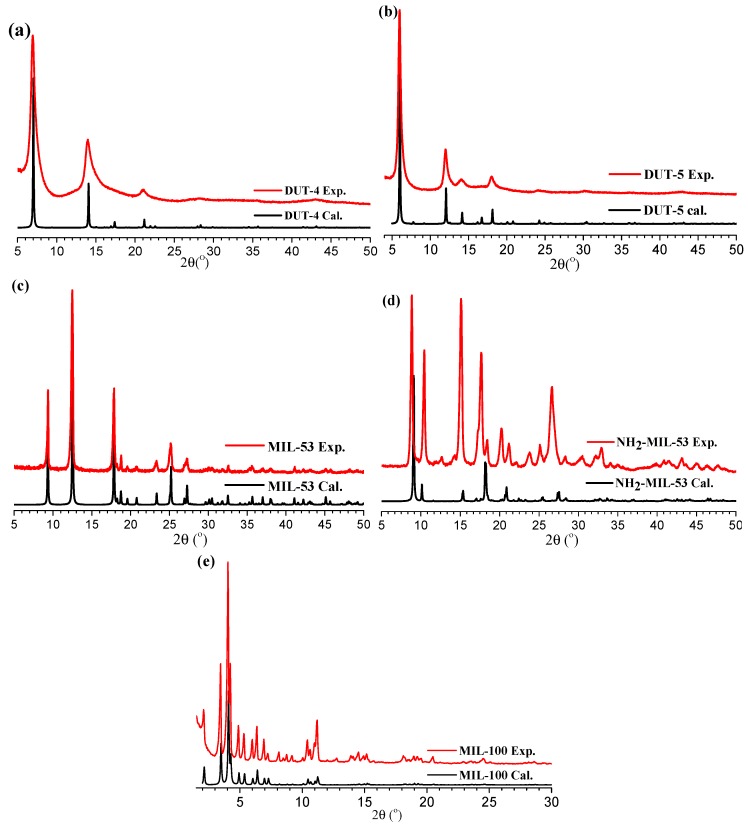
The comparison of PXRD patterns of activated MOFs: (**a**) DUT-4; (**b**) DUT-5; (**c**) MIL-53; (**d**) NH_2_-MIL-53; and (**e**) MIL-100 with their calculated PXRD patterns.

The phase purity of MW-synthesized MOFs was initially checked by PXRD measurements and FT-IR (Figure S2, Supplementary Materials) data. Comparing the PXRD patterns of activated samples of Al-MOFs with their calculated PXRD patterns [1,2,3,4] confirms their authenticity (see Figure 1). The thermal stability of the MW-synthesized MOFs was studied by thermogravimetric analyses (Figure S3, Supplementary Materials). As expected, all MOFs exhibited very high thermal stability which is similar to that of their literature reports [1,2,3,4]. The coordination environments around Al atoms and the porous framework structures of all five nanoporous MOFs are given in Figure S1 (Supplementary Materials).

**Figure 2:**
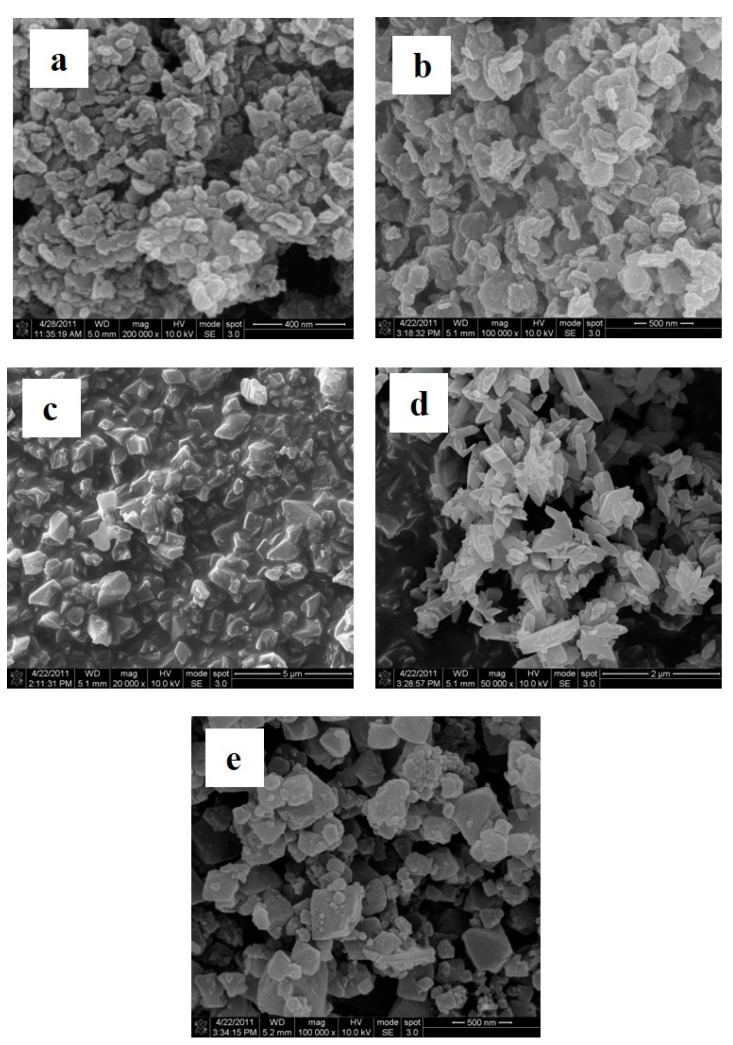
The SEM images for MW-synthesized MOFs of: (**a**) DUT-4; (**b**) DUT-5; (**c**) MIL-53; (**d**) NH_2_-MIL-53; and (**e**) MIL-100.

We have further analyzed the morphology and particle size of the MW-synthesized MOFs using SEM and particle size analyzer respectively. The SEM images (Figure 2) showed that the MW-synthesized Al-MOFs have similar morphology to their corresponding literature reports [1,2,3,4], but are smaller in crystal size. The particle size analyzer data (Table 1) has further displayed that the particles of the MW-synthesized Al-MOFs are much smaller (approximately 3–5 times) than that of the MOFs synthesized under conventional hydrothermal (CE) method. The MW synthesis brought out fast MOF growth and small particle size which are due to a fast nuclei crystallization during the microwave reactions [28].

### 3.3. Nitrogen Gas Sorption Properties

**Figure 3:**
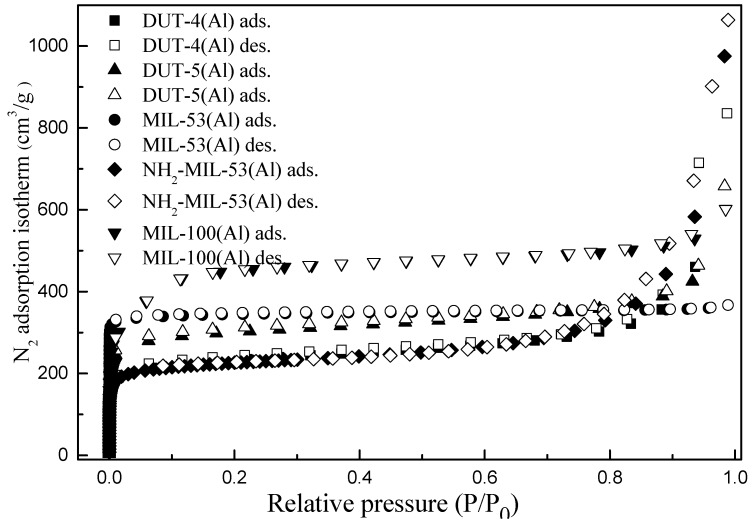
The N_2_ gas sorption isotherms for: (**a**) DUT-4; (**b**) DUT-5; (**c**) MIL-53; (**d**) NH_2_-MIL-53; and (**e**) MIL-100.

The porosity and surface area of the activated samples of our MW-synthesized MOFs were analyzed by nitrogen gas sorption measurements at 77 K (Figure 3). The N_2_ sorption isotherms of the MW-synthesized Al-MOFs displayed permanent microporosity, which is verified by the reversible type I adsorption isotherms [29]. The initial steep step of all of the Al-MOFs increased in the volumetric uptake in the low pressure region followed by a quick saturation step which indicates their microporous nature of all the MOFs. All the sorption isotherms of this Al-MOF series showed the N_2_ gas sorption without hysteresis, indicating the high microporosity of the structure and high stability of the framework. The pore size distribution results of all the MOFs obtained using the density functional theory (DFT) method are given in Figure S4 (Supplementary Materials), which further confirms their microporous nature.

In addition, the comparison of porosity data of our MW-synthesized MOFs with existing literature data is given in Table 2. The data in Table 2 clearly indicated that the MOFs synthesized under MW conditions have better pore size and pore volume with slightly lesser surface area (except MIL-53(Al) case which has better surface area) than that of the same MOFs synthesized under CE conditions. Overall, the porosity data obtained from N_2_ gas adsorption measurements of our MW-assisted synthesized MOFs were comparable well with the same Al-MOFs synthesized under CE conditions previously, which once again confirms the good quality of the synthesized MOF materials under MW conditions.

### 3.4. Catalytic Activity of Synthesized MOFs in Sulfoxidation Reaction

**Scheme 1:**
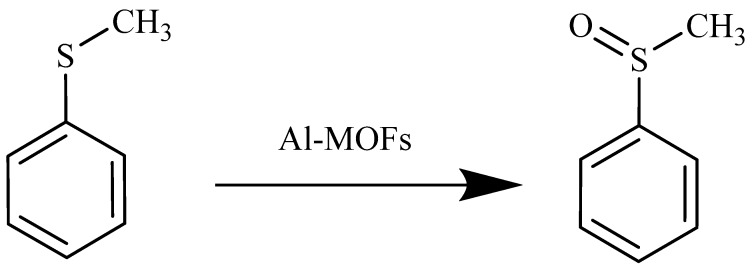
The sulfoxidation of reaction thioanisole to methyl phenyl sulfoxide catalyzed by Al-MOFs.

To evaluate the application prospective of our synthesized MOFs, all of them have been tested for their catalytic activity in the sulfoxidation reaction of sulfides. As we have already seen in the introduction section, although the sulfoxidation reaction of sulfides has been studied extensively, the designing of highly effective and selective catalysts is still desirable and the MOF based catalytic system are relatively new for this reaction. Hence, we have examined the catalytic properties of the synthesized nanoporous Al-MOFs in the sulfoxidation reaction of methyl phenyl sulfide (Scheme 1).

The oxidation of thioanisole was performed in ACN using 1 mol % loading of Al-MOF catalyst and 1 equiv. of H_2_O_2_ (35% in water) at room temperature. We have examined different stoichiometric ratios for the oxidizing agent. One equivalent of H_2_O_2_ (35% in water) was found most useful for maximum conversion. Interestingly, only first oxidation product, methyl phenyl sulfoxide has been observed with high yields in most of the cases (Table 3). A very less amount of second oxidation product, methyl phenyl sulfone along with 94% methyl phenyl sulfoxide conversion (more than 98% selectivity) was observed in DUT-4 and DUT-5 catalyzed reactions after 72 h (Table 3, entries 1 and 2). It is to be noted that the blank test, which was also performed by running the reaction without a catalyst (Table 3, entry 6), showed very less amount of the product, which ensures the catalytic activity of our synthesized MOFs.

Since the present sulfoxidation reaction under room temperature conditions took long time (3 days) to complete, the same reaction has been carried out with 60 °C temperature conditions and the results are given in Table 4. The results revealed that the increase in reaction temperature from room temperature to 60 °C drastically increases the rate of the reaction and the reaction completed in 7 h with very good conversion and selectivity. Further, it is exciting to note that the catalysts, MIL-53 and NH_2_-MIL-53 led sulfoxide (methyl phenyl sulfoxide) product selectively with no traces of sulfone product. Although a small traces of methyl phenyl sulfone product has been observed in the remaining three catalytic cases (DUT-4, DUT-5, and MIL-100), the overall conversion and selectivity of the sulfoxide were found to be more than 95% (Table 4, entries 1, 2 and 5) which can be comparable with reported MOF based sulfoxidation catalysts in the literature [25,26].

It is known that the aluminum MOFs reported in this article are labile to form the coordinately unsaturated aluminum centers in the MOF architecture which is believed to be responsible for the observed high catalytic activity and selectivity of these MOFs. In addition, the microporosity of all these Al-MOFs are also supposed to have a positive influence on their selective sulfoxidation catalytic ability. The mechanism for the MOF-based sulfoxidation catalysts has already been proposed and involves the formation of a peroxo-metal active species, in which the peroxo group is coordinated to the unsaturated metal center as intermediate. Then, nucleophilic attack of the sulfide occurs on the intermediate and a subsequent concerted oxygen transfer forms the resulting sulfoxide compound and H_2_O [30]. A comparison of the PXRD pattern of all the MOFs before and after catalysis showed that the structure remains unchanged which proves the stability of all the MOFs during catalysis (Figure S15, Supplementary Materials). Overall, the catalytic activity evaluation results of MW-assisted greenly synthesized microporous Al-MOFs on the representative reaction of sulfoxidation of thioanisole suggested that they have great potential to catalyze the sulfide into sulfoxide selectively with low catalyst concentration (1 mol %), and less oxidant (1 equiv. of 35% H_2_O_2_ in water) usage under mild reaction conditions (25 °C/72 h or 60 °C/7 h).

## 4. Conclusions

Five nanoporous Al–MOFs, DUT-4, DUT-5, MIL-53, NH_2_-MIL-53 and MIL-100, have been successfully prepared through a facile and environmentally friendly MW-assisted hydrothermal reactions. The MW-assisted method drastically reduced the reaction time from few days to less than 2 h, and the MOFs have a smaller particle size with a relatively uniform particle size distribution with better yield and porous properties. Further, an economic and simple process for the synthesis of sulfoxides selectively by oxidation of sulfides with 35% aq. H_2_O_2_ over our MW-assisted greenly synthesized MOFs as heterogeneous catalyst was also revealed (mostly, the overall conversion and selectivity of the sulfoxide was more than 95%).

## Figures and Tables

**Table 1:** Particle size values of Al-MOFs synthesized by MW and CE method.

S. No.	Al-MOF	Dispersion Medium	Particle Size (nm) (MW/CE)
1.	DUT-4	Water	340/816
2.	DUT-5	Water	278/1528
3.	MIL-53	Water	390/806
4.	NH_2_-MIL-53	Water	477/1607
5.	MIL-100	Water	592/2407

**Table 2:** Comparison of porosity data our MW-synthesized MOFs with their corresponding literature.

Al-MOF	Reaction Cond. Temp./Time	Pore Size (Å)	Pore Volume (cm^3^/g)	Surface Area (m^2^·g^−1^) (BET/Langmuir)	Ref.
DUT-4	120 °C/24 h	8.5	0.68	1308/1996	[1]
	120 °C/52 min	10.2, 15.6	0.75	1184/1340	CW
DUT-5	120 °C/24 h	11.1	0.81	1613/2335	[1]
	120 °C/83 min	12.4	0.83	1157/1305	CW
MIL-53	220 °C/72 h	8.5	0.68	1181/1590	[2]
	200 °C/46 min	11.3	0.73	1403/1511	CW
NH_2_-MIL-53	130 °C/48-144 h	-	0.48	811/-	[3]
	200 °C/46 min	8.8, 13.8	1.0	865/1013	CW
MIL-100	210 °C/72 h	-	0.82	2152/2919	[4]
	200 °C/83 min	8.6, 11.8, 20.0	0.85	1701/1967	CW

CW = Current Work.

**Table 3:** Summary of selective oxidation of methyl phenyl sulfide to methyl phenyl sulfoxide catalyzed by Al-MOFs at room temperature.

Entry	Catalyst	Conversion % ^a^			% Selectivity of Sulfoxide for 72 h ^a^
		24 h	48 h	72 h	
1	DUT-4	55	88	94 ^b^	98.8
2	DUT-5	49	83	94 ^b^	98.2
3	MIL-53	59	80	97	>99
4	NH_2_-MIL-53	38	81	86	>99
5	MIL-100	54	81	90	>99
6	-	7	9	13	-

^a^ Determined by ^1^H NMR(300 MHz) (Figures S5–S9, Supplementary Materials); ^b^ found second oxidation product peak by ^1^H NMR.

**Table 4:** Summary of selective oxidation of methyl phenyl sulfide to methyl phenyl sulfoxide catalyzed by Al-MOFs at 60 °C.

Entry	Catalyst	Conversion % ^a^					% Selectivity of Sulfoxide for 7 h ^a^
		3 h	4 h	5 h	6 h	7 h	
1	DUT-4	78	86	92	92 ^b^	98 ^b^	97.7
2	DUT-5	83 ^b^	92 ^b^	93 ^b^	94 ^b^	95 ^b^	95.5
3	MIL-53	75	87	87	95	99	>99
4	NH_2_-MIL-53	76	86	90	94	96	>99
5	MIL-100	87	94	95 ^b^	95 ^b^	99 ^b^	99

^a^ Determined by ^1^H NMR(300 MHz) (Figures S10–S14, Supplementary Materials); ^b^ found second oxidation product peak by ^1^H NMR.

## Supplementary Materials

The following are available online at www.mdpi.com/2073-4360/9/10/498/s1. Figure S1: The coordination environments of Al atoms (left) and porous structures (right) of (a) DUT-4(Al), (b) DUT-5(Al), (c) MIL-53(Al), (d) NH_2_-MIL-53(Al) and (e) MIL-100(Al); Figure S2: The FT-IR spectra of activated samples of DUT-4(Al), DUT-5(Al), MIL-53(Al), NH_2_-MIL-53(Al), and MIL-100(Al); Figure S3: The thermogravimetric analysis curve of activated samples of DUT-4(Al), DUT-5(Al), MIL-53(Al) NH_2_-MIL-53(Al) and MIL-100(Al); Figure S4: The DFT pore size distribution for DUT-4(Al), DUT-5(Al), MIL-53(Al), NH_2_-MIL-53(Al), and MIL-100(Al); Figure S5: ^1^H NMR spectrum of catalytic result for DUT-4(Al) at room temperature for 24 (a), 48 (b) and 72 h (c); Figure S6: ^1^H-NMR spectrum of catalytic result for DUT-5(Al) at room temperature for 24 (a), 48 (b) and 72 h (c); Figure S7: ^1^H NMR spectrum of catalytic result for MIL-53(Al) at room temperature for 24 (a), 48 (b) and 72 h (c); Figure S8: ^1^H NMR spectrum of catalytic result for NH_2_-MIL-53(Al) at room temperature for 24 (a), 48 (b) and 72 h (c); Figure S9: ^1^H-NMR spectrum of catalytic result for MIL-100(Al) at room temperature for 24 (a), 48 (b) and 72 h (c); Figure S10: ^1^H NMR spectrum of catalytic result for DUT-4(Al) at 60 °C for 3 (a), 4 (b), 5 (c), 6 (d) and 7 h (e); Figure S11: ^1^H NMR spectrum of catalytic result for DUT-5(Al) at 60 °C for 3 (a), 4 (b), 5 (c), 6 (d) and 7 h (e); Figure S12: ^1^H NMR spectrum of catalytic result for MIL-53(Al) at 60 °C for 3 (a), 4 (b), 5 (c), 6 (d) and 7 h (e); Figure S13: ^1^H NMR spectrum of catalytic result for NH_2_-MIL-53(Al) at 60 °C for 3 (a), 4 (b), 5 (c), 6 (d) and 7 h (e); Figure S14: ^1^H NMR spectrum of catalytic result for MIL-100(Al) at 60 ºC for 3 (a), 4 (b), 5 (c), 6 (d) and 7 h (e); Figure S15: The comparison of PXRD patterns of (a) DUT-4(Al), (b) DUT-5(Al), (c) MIL-53(Al), (d) NH_2_-MIL-53(Al), and (e) MIL-100(Al) before and after catalysis.

## References

[1] Senkovska I., Hoffmann F., Fröba M., Getzschmann J., Böhlmann W., Kaskel S. (2009). New highly porous aluminium based metal-organic frameworks: Al(OH)(ndc) (ndc = 2,6-naphthalene dicarboxylate) and Al(OH)(bpdc) (bpdc = 4,4′-biphenyl dicarboxylate). Microporous Mesoporous Mater..

[2] Loiseau T., Serre C., Huguenard C., Fink G., Taulelle F., Henry M., Bataille T., Ferey G. (2004). A Rationale for the Large Breathing of the Porous Aluminum Terephthalate (MIL-53) Upon Hydration. Chem. Eur. J..

[3] Kim J., Kim W.Y., Ahn W.S. (2012). Amine-functionalized MIL-53 (Al) for CO_2_/N_2_ separation: Effect of textural properties. Fuel.

[4] Volkringer C., Popov D., Loiseau T., Ferrey G., Burghammer M., Riekel C., Haouas M., Taulelle F. (2005). Synthesis, single-crystal X-ray microdiffraction, and NMR characterizations of the giant pore metal-organic framework aluminum trimesate MIL-100. Chem. Mater..

[5] Liu J., Zhang F., Zou X., Yu G., Zhao N., Fan S., Zhu G. (2013). Environmentally friendly synthesis of highly hydrophobic and stable MIL-53 MOF nanomaterials. Chem. Commun..

[6] Stock N. (2014). Metal-organic frameworks: Aluminium-based frameworks. Encyclopedia of Inorganic and Bioinorganic Chemistry.

[7] Trung T.K., Trens P., Tanchoux N., Bourrelly S., Llewellyn P.L., Loera-Serna S., Serre C., Loiseau T., Fajula F., Ferey G. (2008). Hydrocarbon adsorption in the flexible metal organic frameworks MIL-53 (Al, Cr). J. Am. Chem. Soc..

[8] Lohe M.R., Gedrich K., Freudenberg T., Kockrick E., Dellmann T., Kaskel S. (2011). Heating and separation using nanomagnet-functionalized metal-organic frameworks. Chem. Commun..

[9] Qiu M., Guan Q., Li W. (2016). Controllable Assembly of Al-MIL-100 via an Inducing Occupied Effect and Its Selective Adsorption Activity. Cryst. Growth Des..

[10] Lo S.-H., Chien C.-H., Lai Y.-L., Yang C.-C., Lee J.-J., Senthil Raja D., Lin C.-H. (2013). A mesoporous aluminium metal-organic framework with 3 nm open pores. J. Mater. Chem. A.

[11] Liu W.-L., Lo S.-H., Singco B., Yang C.-C., Huang H.-Y., Lin C.-H. (2013). Novel trypsin-FITC@ MOF bioreactor efficiently catalyzes protein digestion. J. Mater. Chem. B.

[12] Shih Y.-H., Fu C.-P., Liu W.-L., Lin C.-H., Huang H.-Y., Ma S. (2016). Nanoporous Carbons Derived from Metal-Organic Frameworks as Novel Matrices for Surface-Assisted Laser Desorption/Ionization Mass Spectrometry. Small.

[13] Senthil Raja D., Chang I.-H., Jiang Y.-C., Chen H.-T., Lin C.-H. (2015). Enhanced gas sorption properties of a new sulfone functionalized aluminum metal-organic framework: Synthesis, characterization, and DFT studies. Microporous Mesoporous Mater..

[14] Lo S.-H., Senthil Raja D., Chen C.-W., Kang Y.-H., Chen J.-J., Lin C.-H. (2016). Waste polyethylene terephthalate (PET) materials as sustainable precursors for the synthesis of nanoporous MOFs, MIL-47, MIL-53 (Cr, Al, Ga) and MIL-101 (Cr). Dalton Trans..

[15] Stock N., Biswas S. (2012). Synthesis of metal-organic frameworks (MOFs): Routes to various MOF topologies, morphologies, and composites. Chem. Rev..

[16] Khan N.A., Jhung S.H. (2015). Synthesis of metal-organic frameworks (MOFs) with microwave or ultrasound: Rapid reaction, phase-selectivity, and size reduction. Coord. Chem. Rev..

[17] Kappe C.O. (2004). Controlled microwave heating in modern organic synthesis. Angew. Chem. Int. Ed..

[18] Klinowski J., Paz F.A.A., Silva P., Rocha J. (2011). Microwave-assisted synthesis of metal-organic frameworks. Dalton Trans..

[19] Choi J.-S., Son W.-J., Kim J., Ahn W.-S. (2008). Metal-organic framework MOF-5 prepared by microwave heating: Factors to be considered. Microporous Mesoporous Mater..

[20] Seo Y.-K., Hundal G., Jang I.T., Hwang Y.K., Jun C.-H., Chang J.-S. (2009). Microwave synthesis of hybrid inorganic-organic materials including porous Cu_3_(BTC)_2_ from Cu(II)-trimesate mixture. Microporous Mesoporous Mater..

[21] Taddei M., Dau P.V., Cohen S.M., Ranocchiari M., van Bokhoven J.A., Costantino F., Sabatini S., Vivani R. (2015). Efficient microwave assisted synthesis of metal-organic framework UiO-66: Optimization and scale up. Dalton Trans..

[22] Yeh C.-T., Lin W.-C., Lo S.-H., Kao C.-C., Lin C.-H., Yang C.-C. (2012). Microwave synthesis and gas sorption of calcium and strontium metal-organic frameworks with high thermal stability. CrystEngComm.

[23] Lane B.S., Burgess K. (2003). Metal-catalyzed epoxidations of alkenes with hydrogen peroxide. Chem. Rev..

[24] Shaabani A., Rezayan A.H. (2007). Silica sulfuric acid promoted selective oxidation of sulfides to sulfoxides or sulfones in the presence of aqueous H_2_O_2_. Catal. Commun..

[25] Zhu C., Chen X., Yang Z., Du X., Liu Y., Cui Y. (2013). Chiral microporous Ti (salan)-based metal-organic frameworks for asymmetric sulfoxidation. Chem. Commun..

[26] Xie M.-H., Yang X.-L., Zou C., Wu C.-D. (2011). A SnIV-porphyrin-based metal-organic framework for the selective photo-oxygenation of phenol and sulphides. Inorg. Chem..

[27] Liu J., Chen L., Cui H., Zhang J., Zhang L., Su C.-Y. (2014). Applications of metal-organic frameworks in heterogeneous supramolecular catalysis. Chem. Soc. Rev..

[28] Jhung S.H., Jin T., Hwang Y.K., Chang J.-S. (2007). Microwave effect in the fast synthesis of microporous materials: which stage between nucleation and crystal growth is accelerated by microwave irradiation?. Chem. Eur. J..

[29] Sing K.D., Everett D.H., Haul R., Moscou L., Pierotti R., Rouquerol J., Siemieniewska T. (1985). Commission on colloid and surface chemistry including catalysis. Pure Appl. Chem..

[30] Tanaka K., Kubo K., Iida K., Otani K.-I., Murase T., Yanamoto D., Shiro M. (2013). Asymmetric Catalytic Sulfoxidation with H_2_O_2_ using Chiral Copper Metal-Organic Framework Crystals. Asian J. Org. Chem..

