# Supplementary material for: Microwave-Assisted Synthesis of Nanoporous Aluminum-Based Coordination Polymers as Catalysts for Selective Sulfoxidation Reaction
Source: Polymers (Basel). 2017 Oct 11;9(10):498. doi: 10.3390/polym9100498 (PMC6418783; doi:10.3390/polym9100498)
Supplement: Supplementary file 1 [file polymers-09-00498-s001.pdf]

Supporting Information

Microwave-Assisted Synthesis of Aluminium MOFs as Catalysts for Selective Sulfoxidation Reaction

Madhan Vinu, Wei-Cheng Lin, Duraisamy Senthil Raja, Jeng-Liang Han* and Chia-Her Lin*

Department of Chemistry, Chung Yuan Christian University, Chung Li, Taoyuan, Taiwan 32023.

Correspondence: jlhan@cycu.edu.tw (J.-L.H.); chiaher@cycu.edu.tw (C.-H.L.)

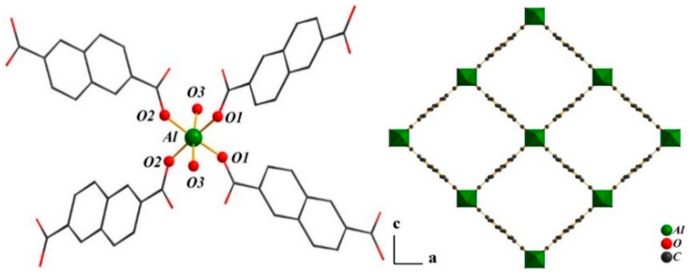

(a)

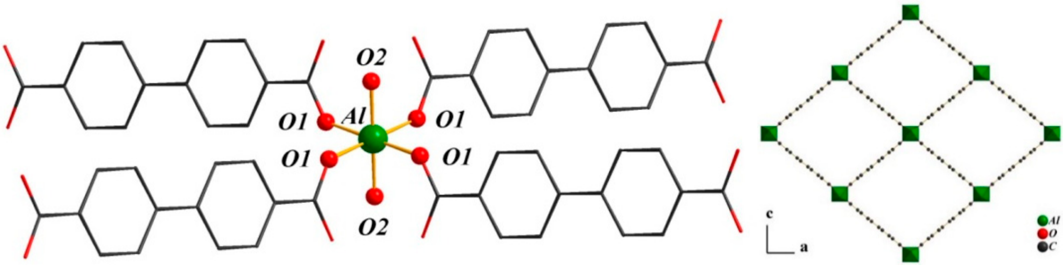

(b)

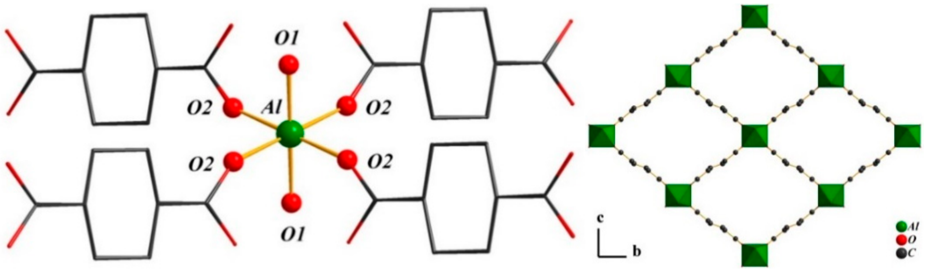

(c)

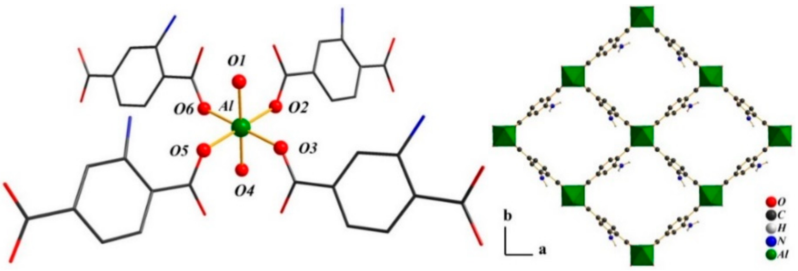

(d)

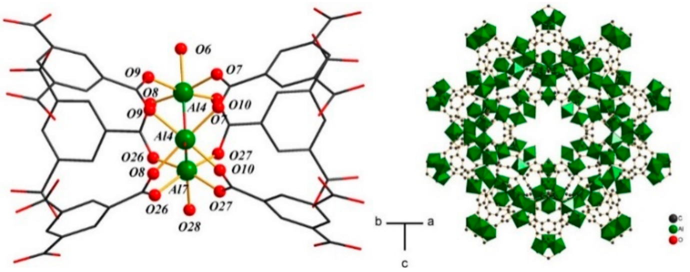

(e)

Figure S1. The coordination environments of Al atoms (left) and porous structures (right) of (a) DUT-4(Al), (b) DUT-5(Al), (c) MIL-53(Al), (d) NH_2-MIL-53(Al) and (e) MIL-100(Al).

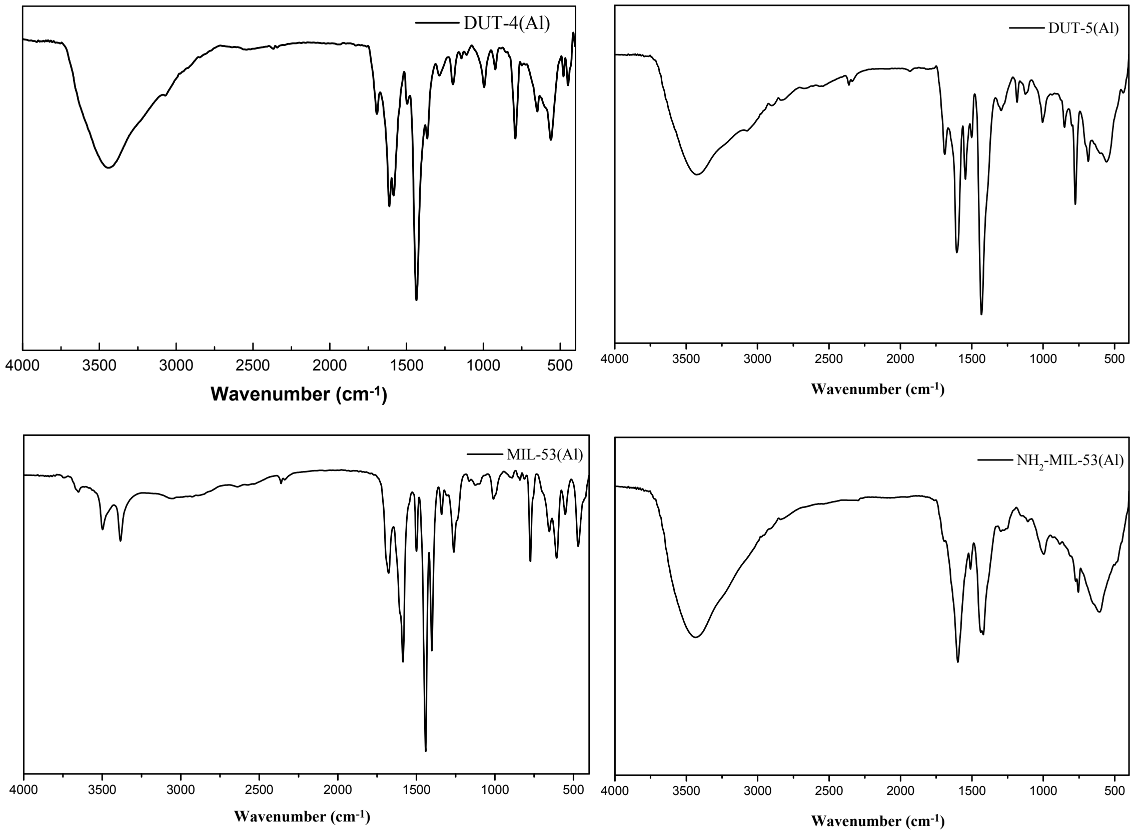

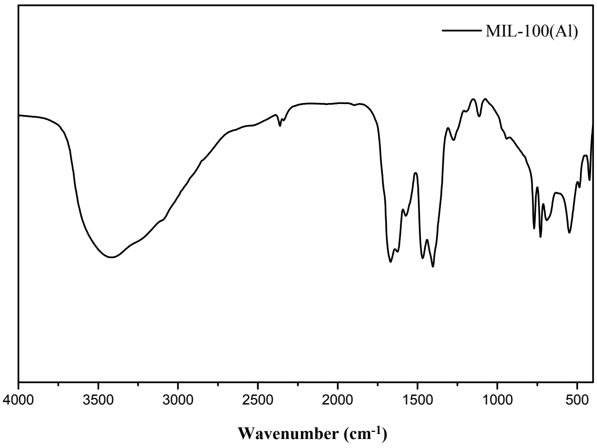

Figure S2. The FT-IR spectra of activated samples of DUT-4(Al), DUT-5(Al), MIL-53(Al), NH_2-MIL-53(Al), and MIL-100(Al).

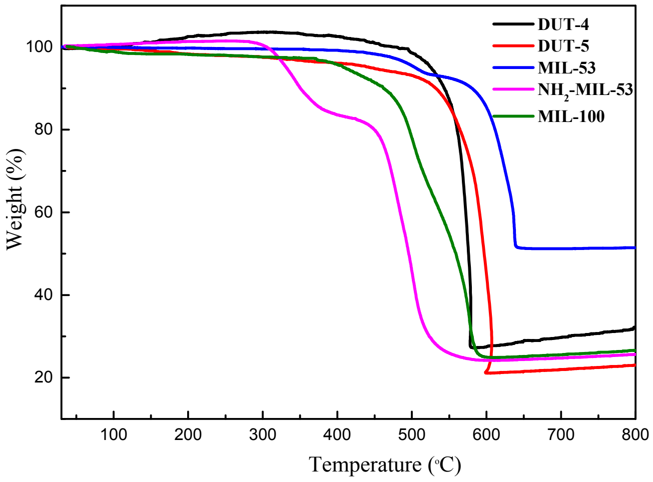

Figure S3. The thermogravimetric analysis curve of activated samples of DUT-4(Al), DUT-5(Al), MIL-53(Al) NH_2-MIL-53(Al) and MIL-100(Al).

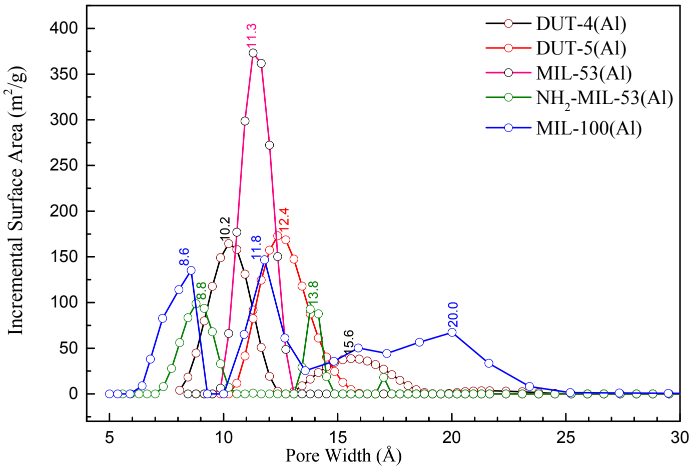

Figure S4. The DFT pore size distribution for DUT-4(Al), DUT-5(Al), MIL-53(Al), NH_2-MIL-53(Al), and MIL-100(Al).

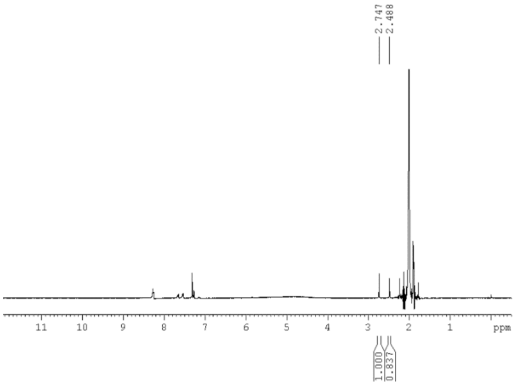

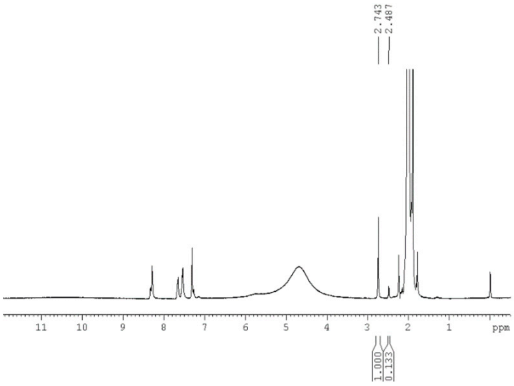

(a) **(b)**

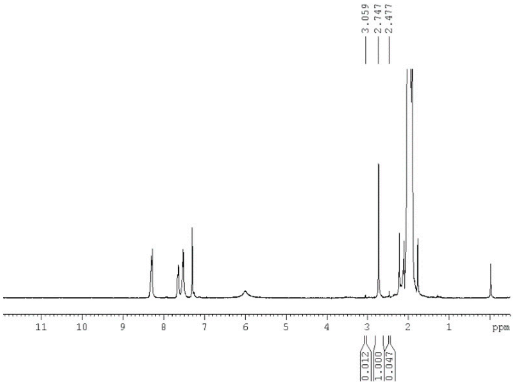

(c)

Figure S5. ^{1}H NMR spectrum of catalytic result for DUT-4(Al) at room temperature for 24 (a), 48 (b) and 72 h (c).

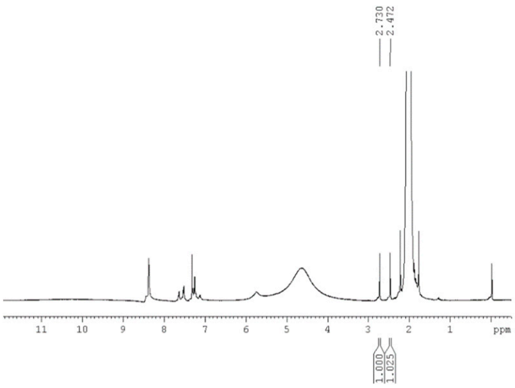

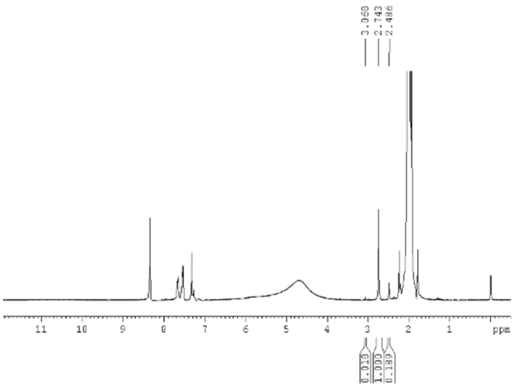

(a) **(b)**

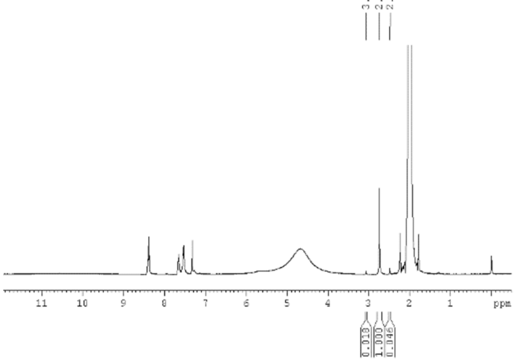

(c)

Figure S6. ^{1}H-NMR spectrum of catalytic result for DUT-5(Al) at room temperature for 24 (a), 48 (b) and 72 h (c).

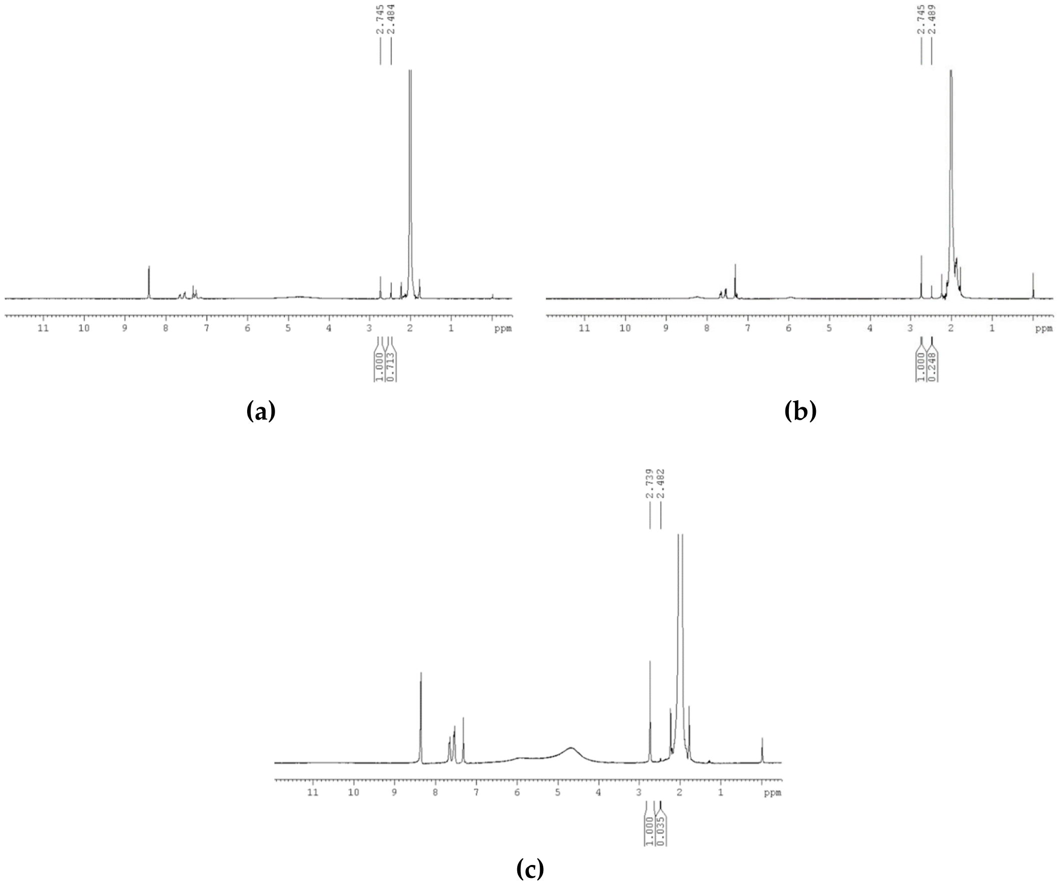

Figure S7. ^{1}H NMR spectrum of catalytic result for MIL-53(Al) at room temperature for 24 (a), 48 (b) and 72 h (c).

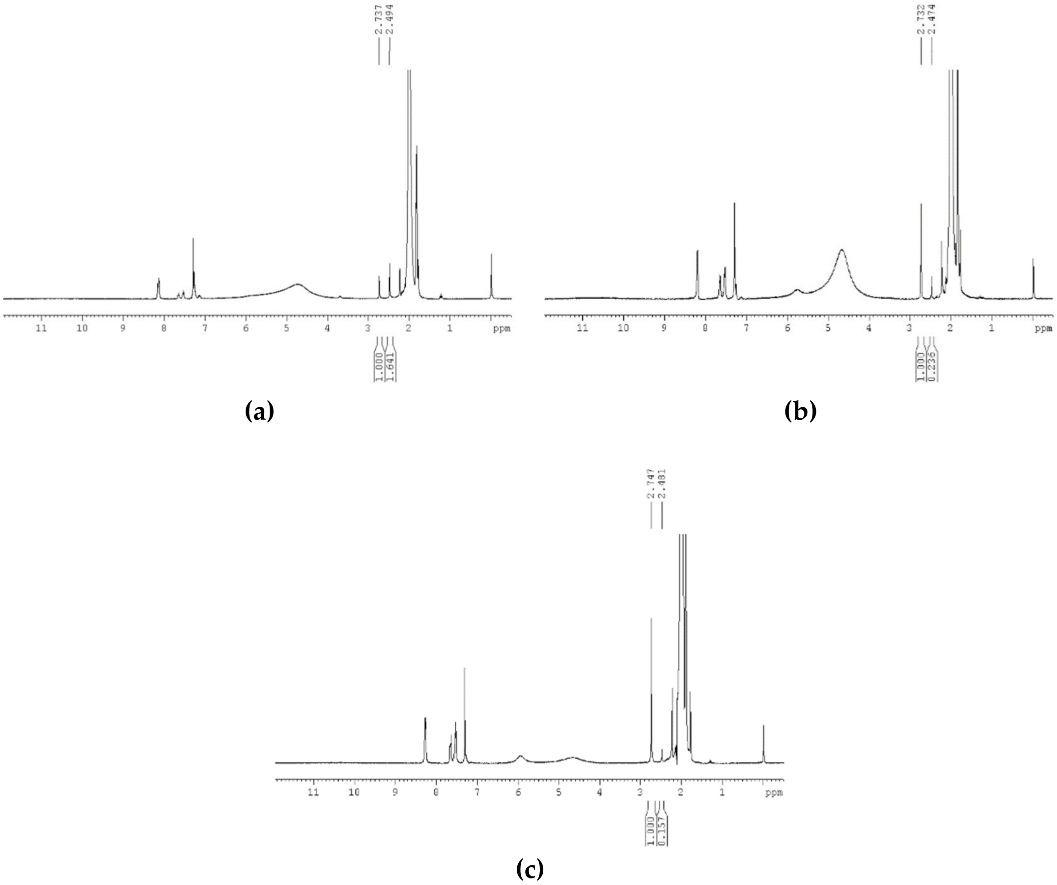

Figure S8. ^{1}H NMR spectrum of catalytic result for NH_2-MIL-53(Al) at room temperature for 24 (a), 48 (b) and 72 h (c).

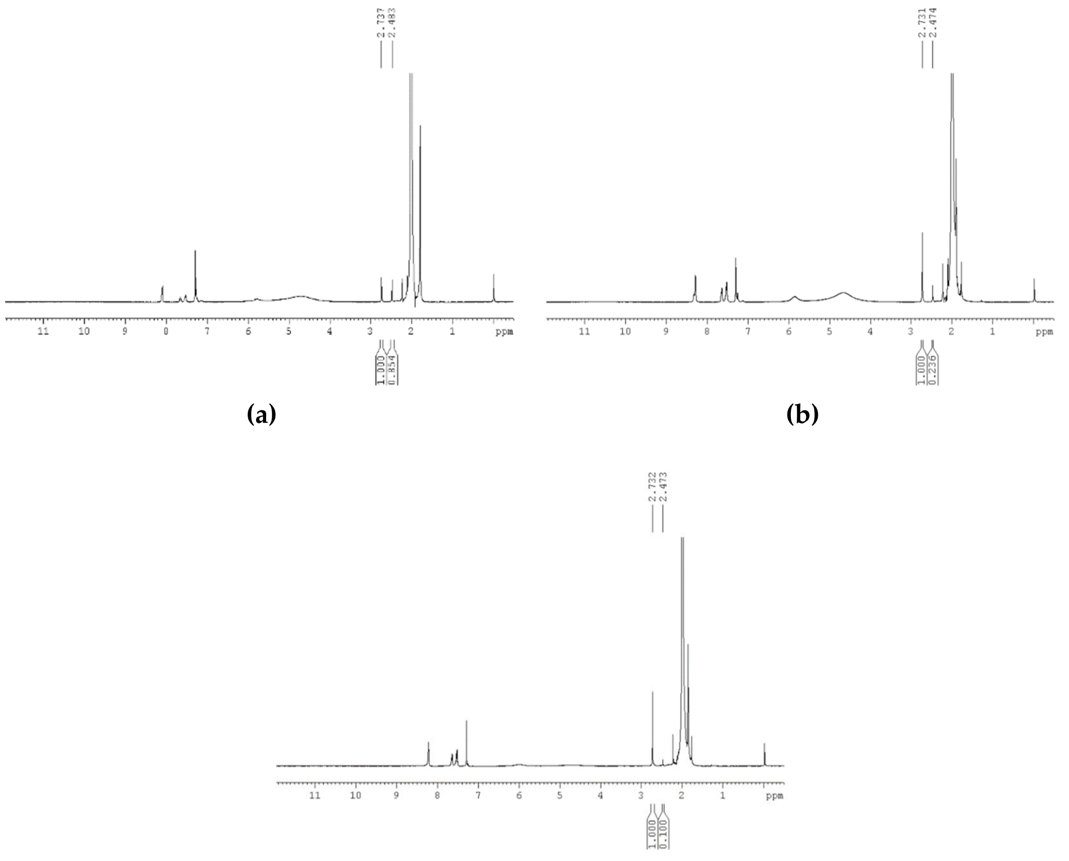

(a) (b)

(c)

Figure S9. ^{1}H-NMR spectrum of catalytic result for MIL-100(Al) at room temperature for 24 (a), 48 (b) and 72 h (c).

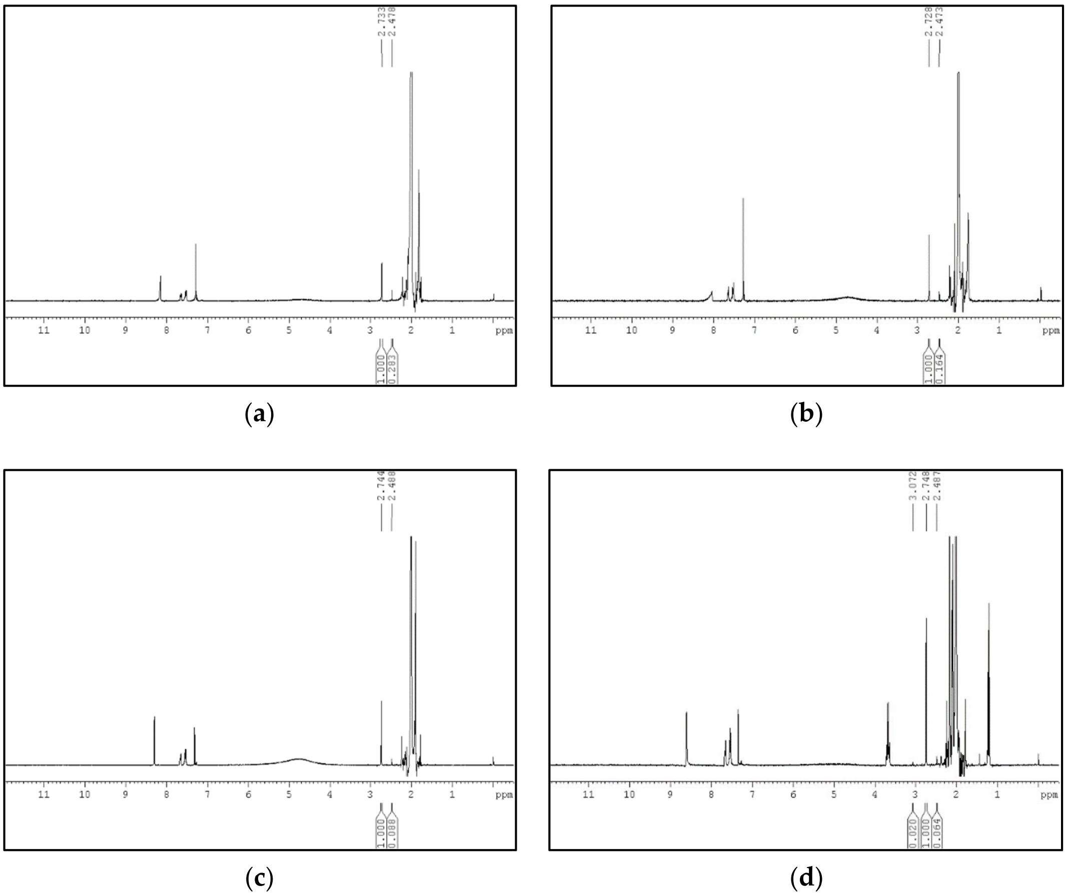

(a) (b)

(c) (d)

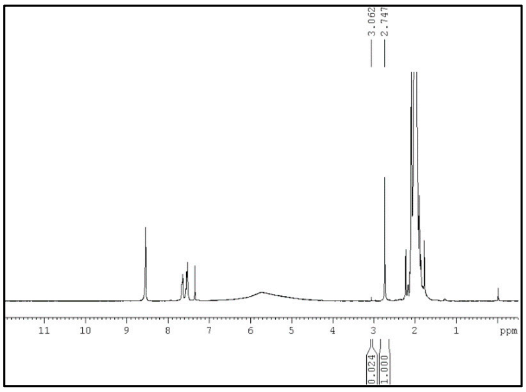

(**e**)

Figure S10. ^{1}H NMR spectrum of catalytic result for DUT-4(Al) at 60 ºC for 3 (a), 4 (b), 5 (c), 6 (d) and 7 h (e).

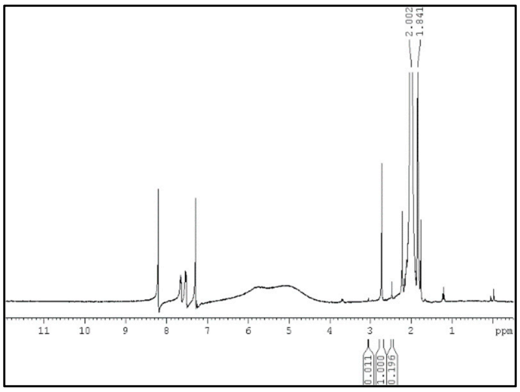

(**a**)

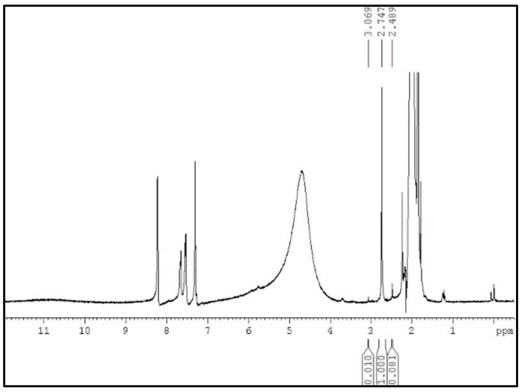

(**b**)

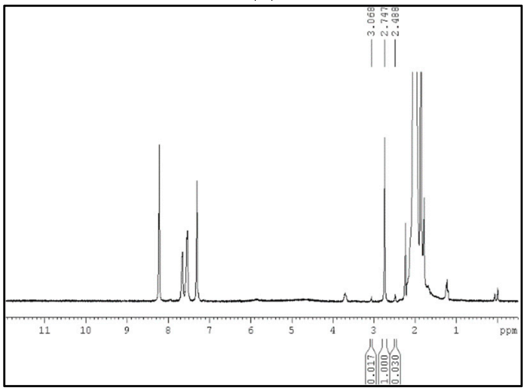

(**c**)

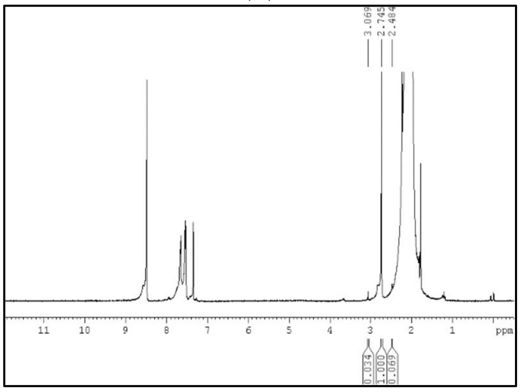

(**d**)

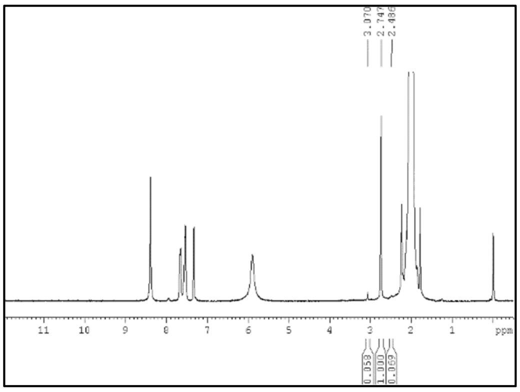

(**e**)

Figure S11. ^{1}H NMR spectrum of catalytic result for DUT-5(Al) at 60 ºC for 3 (a), 4 (b), 5 (c), 6 (d) and 7 h (e).

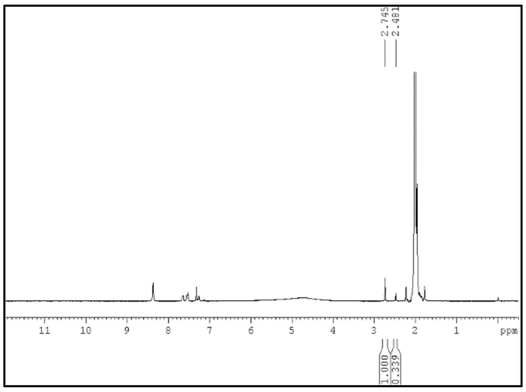

(**a**)

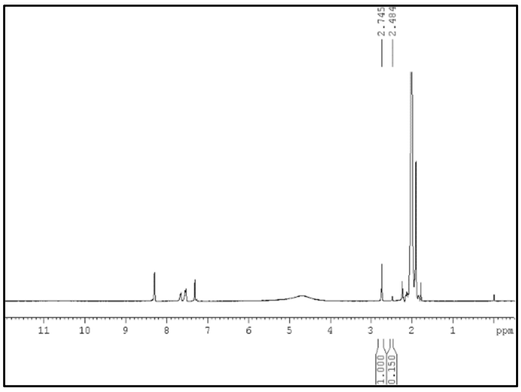

(**b**)

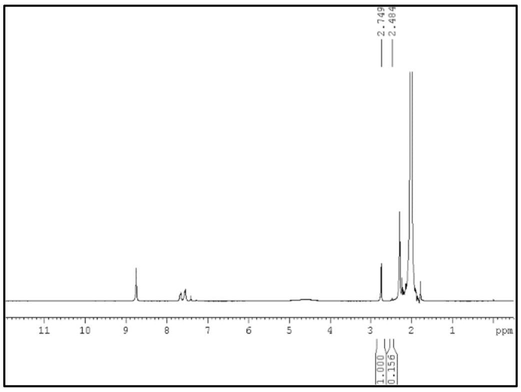

(**c**)

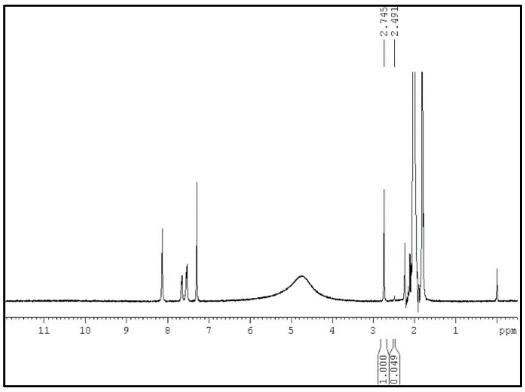

(**d**)

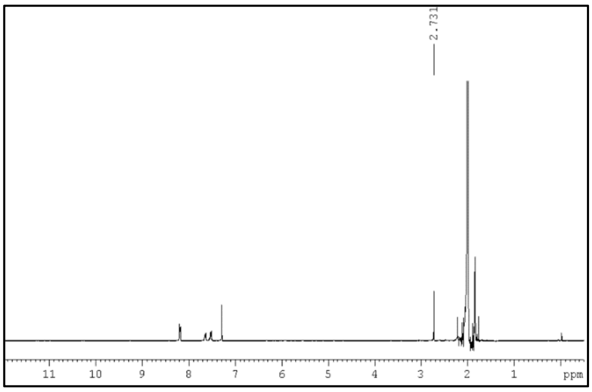

(**e**)

Figure S12. ^{1}H NMR spectrum of catalytic result for MIL-53(Al) at 60 ºC for 3 (a), 4 (b), 5 (c), 6 (d) and 7 h (e).

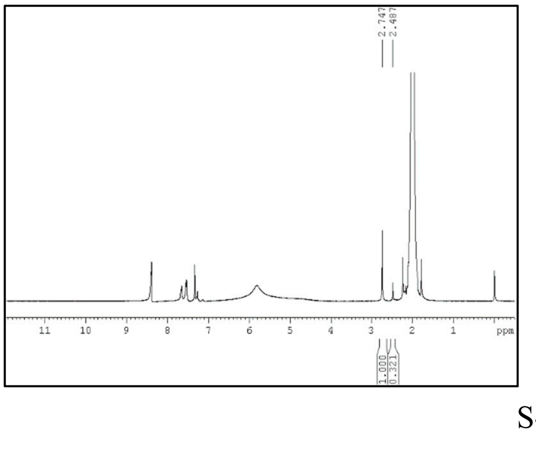

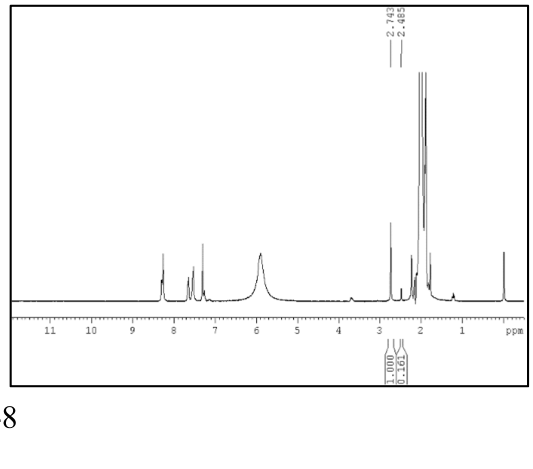

(**a**) (**b**)

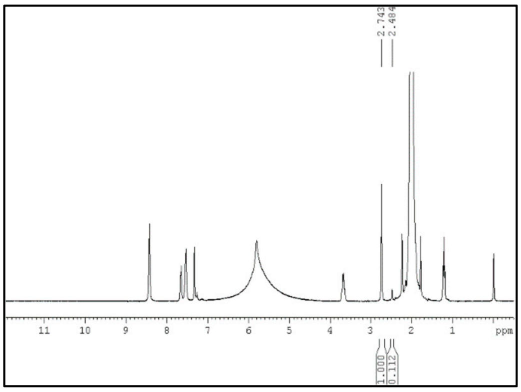

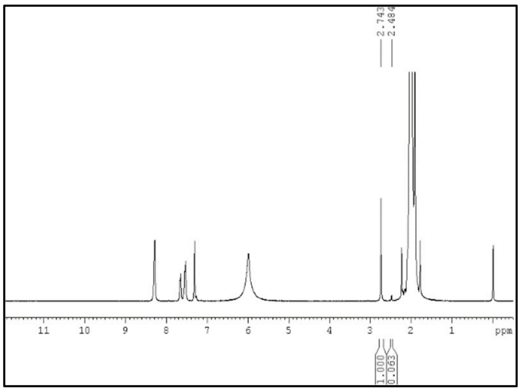

(**c**) (**d**)

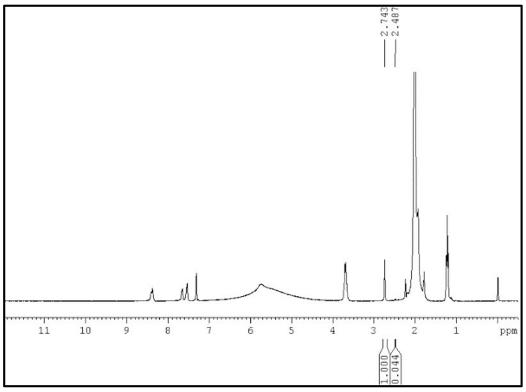

(**e**)

Figure S13. ^{1}H NMR spectrum of catalytic result for NH_2-MIL-53(Al) at 60 ºC for 3 (a), 4 (b), 5 (c), 6 (d) and 7 h (e).

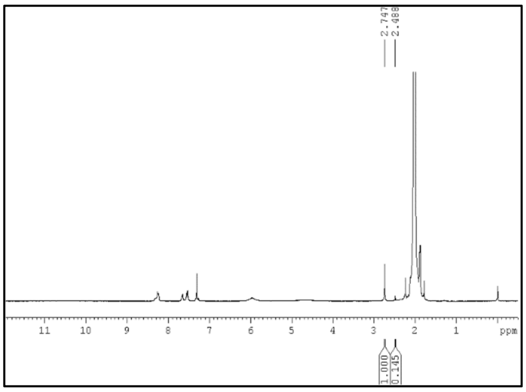

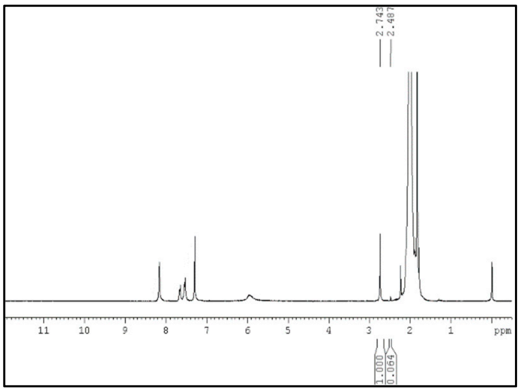

(**a**) (**b**)

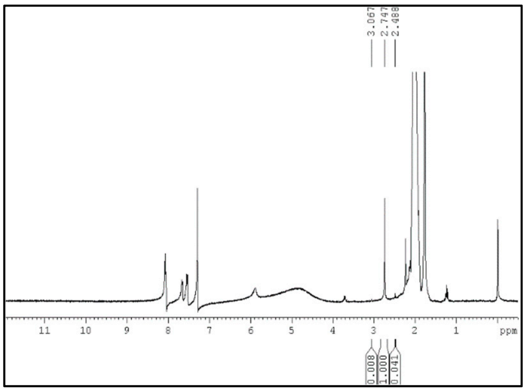

(**c**)

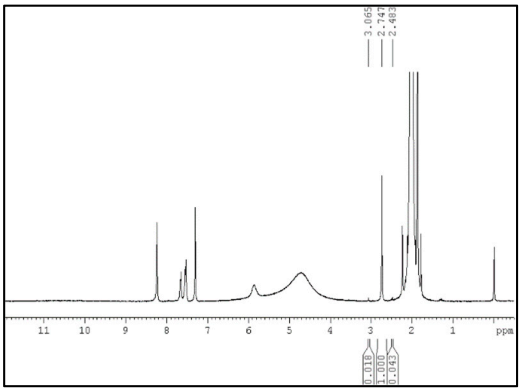

(**d**)

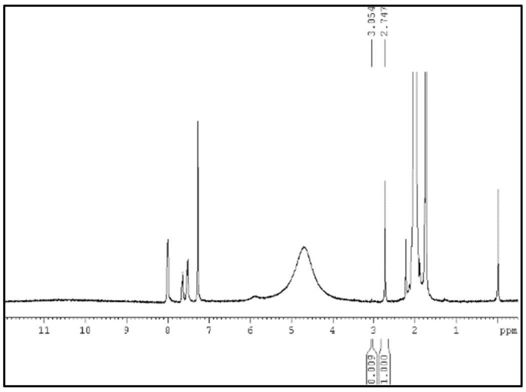

(**e**)

Figure S14. ^{1}H NMR spectrum of catalytic result for MIL-100(Al) at 60 ºC for 3 (a), 4 (b), 5 (c), 6 (d) and 7 h (e).

(**a**) (**b**)

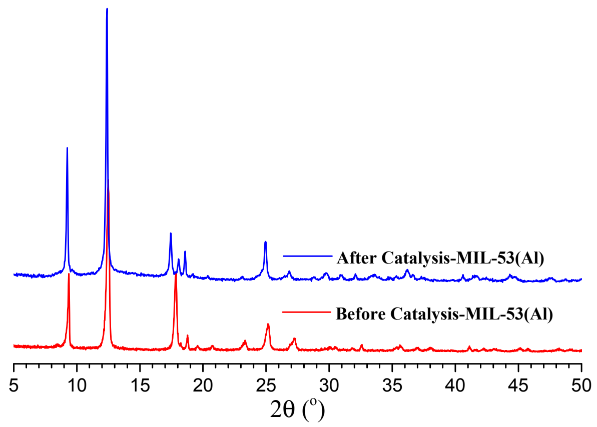

(c)

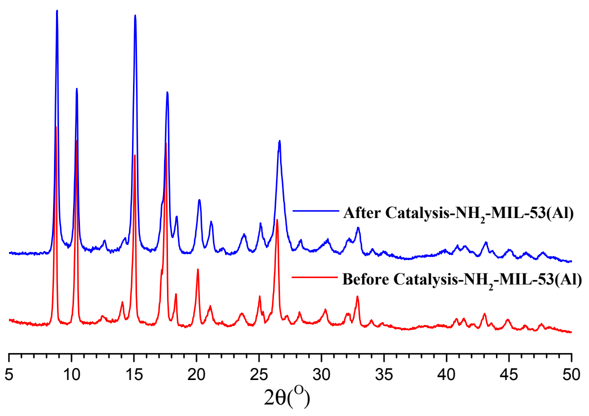

(d)

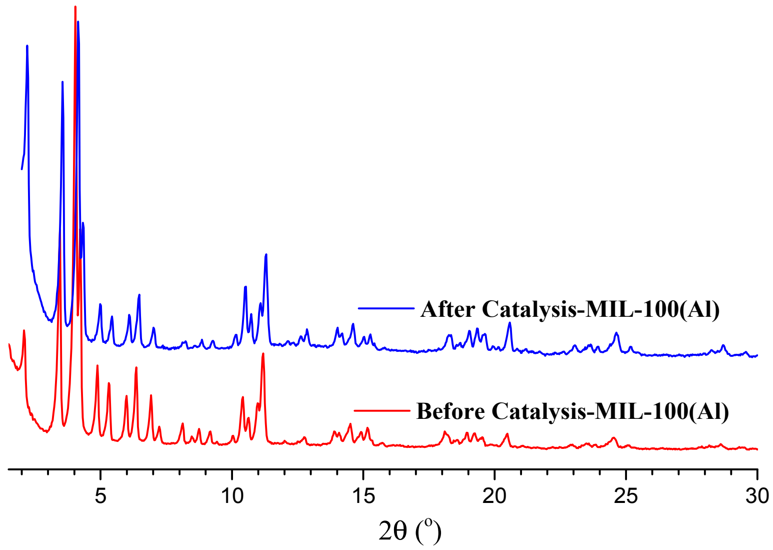

(e)

Figure S15. The comparison of PXRD patterns of (a) DUT-4(Al), (b) DUT-5(Al), (c) MIL-53(Al), (d) NH_2-MIL-53(Al), and (e) MIL-100(Al) before and after catalysis.
